# Intraocular lens models: Ecological distribution footprint and usage trends at a large ophthalmology centre

**DOI:** 10.1038/s41433-025-03848-5

**Published:** 2025-05-20

**Authors:** Benjamin Stern, Damien Gatinel, Georges Nicolaos, Alice Grise-Dulac

**Affiliations:** 1 Anterior Segment and Refractive Surgery Department, Rothschild Foundation Hospital, Paris, France; 2 Division of Ophthalmology, Hadassah Medical Center, Faculty of Medicine, Hebrew University of Jerusalem, Jerusalem, Israel; 3 American-European Congress of Ophthalmic Surgery (AECOS) Green Working Group, Paris, France; 4 EyeSustain and SAFIR (Société de l’association française des implants intra-oculaires et de chirurgie réfractive) Sustainability Working Group, Paris, France

**Keywords:** Health services, Health occupations

## Abstract

**Background:**

Operating theatres significantly contribute to hospital’s environmental footprint, underscoring the need to evaluate the ecological impact of transporting surgical products. Intraocular lenses (IOLs), sourced globally, vary in their environmental impact due to differences in packaging and manufacturing. Assessing the carbon footprint of IOL transport and usage can promote sustainability.

**Methods:**

A retrospective analysis of IOL stock data at Rothschild Foundation Hospital, Paris, was conducted to evaluate all IOLs implanted in 2023. To estimate the ecological footprint of IOL transport, the packaging weight of each model was measured, and the EcoTransIT online calculator was used.

**Results:**

In 2023, a total of 13,894 IOLs from 62 different models were implanted by 112 ophthalmic surgeons at our institution. Carbon dioxide (CO_2_) emissions from the transport of IOL models varied from 1.05 to 12.72 kg per 1,000 units, influenced by packaging weight and shipping distances. Packaging volumes ranged from 135 to 917 cm³. Standard monofocal lenses comprised 65.9% of implanted IOLs, followed by monofocal “plus” lenses at 21.6%. Toric, non-toric extended depth of focus (EDOF), and non-toric multifocal lenses were used less frequently, at 7.4%, 3.9%, and 1.3%, respectively.

**Conclusions:**

Significant disparities exist in CO_2_ emissions related to IOL distribution among different IOL models, highlighting the importance of minimising packaging to reduce environmental impact. Standard monofocal lenses remain the predominant choice among surgeons, with increasing adoption of monofocal “plus” lenses. Premium lenses are used sparingly. Optimising packaging could improve storage efficiency and logistics, potentially facilitating greater adoption of premium lenses, particularly toric lenses.

## Introduction

The field of intraocular lenses (IOLs) is rapidly expanding, driven by a dynamic global market that was valued at 4.2 billion dollars in 2023 and is projected to reach 6 billion dollars by 2029 [1]. This growth is fuelled by continuous innovations from major international manufacturers, which regularly introduce new IOL models. While this diversity promotes competition and potentially lowers prices, it also presents challenges for surgeons navigating a wide array of available options [2]. This rapidly changing landscape contrasts with the slow learning curve of surgeons adapting to new implants, often leading them to prefer older IOL models.

Premium IOLs, including toric, extended depth of focus (EDOF), and multifocal lenses, require additional effort from surgeons, such as explaining the available options to patients and placing special orders. They may also incur extra fees for patients and can sometimes rival the surgeon’s fee. While concerns about implanting premium lenses due to issues such as photic phenomena or reduced contrast sensitivity are understandable, some surgeons opt for monofocal lenses simply because they are easier to manage, informing patients that they will likely need glasses post-surgery.

In an attempt to influence surgeons’ choices, manufacturers employ marketing strategies that often emphasise attractive, oversized packaging to enhance the perceived value of their IOLs. However, this approach increases packaging weight and volume, leading to inefficient stock management, increased waste, and higher greenhouse gas (GHG) emissions from transport, thereby expanding the ecological footprint. Moreover, some manufacturers move their production to countries like China or India to reduce costs and enhance competitiveness, although this shift also heightens GHG emissions related to transport. As the healthcare system in the United States contributes to approximately 10% of the country’s total GHG emissions [3], it is crucial to mitigate the significant environmental impact of healthcare practices. Addressing the transport-related CO_2_ emissions associated with healthcare needs is an important aspect of this effort.

This study aims to evaluate the packaging and ecological impact of transport for different models of IOLs and analyse usage trends at a large European ophthalmology centre, that includes both public and private practices.

## Methods

### Retrospective IOL data extraction

Data regarding intraocular lenses (IOLs) used by surgeons at Rothschild Foundation Hospital in Paris during 2023 were retrospectively extracted from the operating room pharmacy. The extraction utilised stock management software that tracked each lens used by any surgeon.

Subsequently, in May 2024, the packaging of IOL models used in 2023 was meticulously weighed using a high-precision AccuWeight IC255 300 g balance with an accuracy of ±0.05%. Prior to each measurement, the balance underwent calibration to ensure maximum accuracy. For each IOL model, the weight was measured for two boxes and averaged. The dimensions of the package boxes were measured using a standard ruler, defining height as the dimension aligned with the main brand name, width as the perpendicular dimension, and depth as the thickness of the box. Additionally, the manufacturing site of production was verified on all boxes.

### Data analysis

Data regarding IOLs were entered into Microsoft Excel® software (Version 2305 Build 16.0.16501.20074) for calculation of descriptive statistics and graphical analysis. Phakic lenses were excluded. Packaging measurements were conducted after the study period, resulting in missing data on weight, size, packaging, and manufacturing site for 220 implants that were either rarely used or not yet used by May 2024. Therefore, the carbon footprint assessment of transport was conducted solely on 13,674 implants (98.4%).

### Carbon footprint of transport assessment

Greenhouse gases (GHGs) are responsible for climate change by trapping heat in the Earth’s atmosphere. The primary GHGs include carbon dioxide (CO₂), methane (CH₄), nitrous oxide (N₂O), and fluorinated gases (e.g. HFCs, PFCs, SF₆). GHG emissions are quantified as carbon footprint equivalents to assess their impact on climate change more effectively.

To estimate the carbon footprint equivalent of transport, we used the EcoTransIT World calculator available online at https://www.ecotransit.org/. This methodology is endorsed by multiple research institutes (IFEU, INFRAS, Fraunhofer) and complies with the ISO 14083 standard. Detailed methodology information is accessible on the website.

Due to the lack of disclosed data from manufacturers, several key assumptions were made. It was assumed that IOLs are shipped directly from the manufacturer to the hospital in a single, fully loaded annual shipment. Additionally, since the specific mode of transport—whether by truck or sea freight—remains unknown, two shipping scenarios were evaluated: a combination of sea freight and truck transport versus truck-only transport. The scenario yielding the lower emissions was selected. This approach represents the minimum possible GHG emissions scenario for transport.

However, in practice, the supply chain is considerably more complex, often involving multiple storage facilities and partially filled truck shipments. Consequently, it is evident that our estimation underrepresents the actual transport-related emissions.

We provide Well-to-Wheel (WtW) emissions, which estimate GHG emissions from fuel production through to transport completion. Since transport emissions are reported in tons, we incorporated the weight equivalent of 1 million IOL units into our calculations. Subsequently, we converted and reported the emissions for 1000 units for clarity and comparison.

## Results

**Fig. 1:**
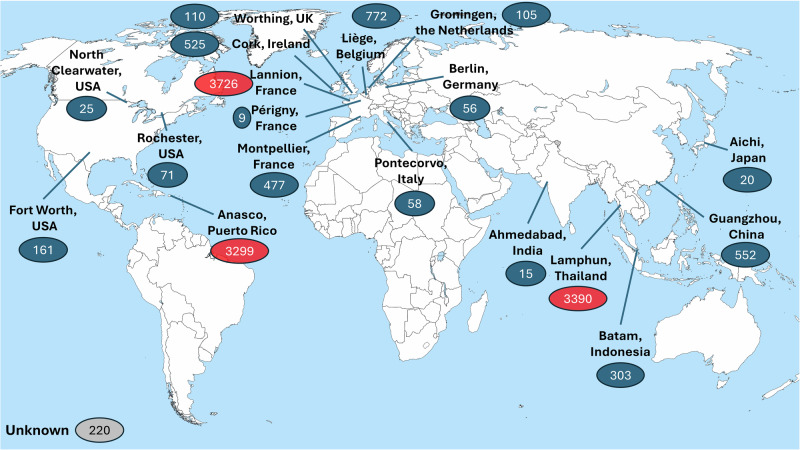
World map illustrating the global distribution of intraocular lens manufacturing sites, indicating the number of lenses used at our institution in 2023 from each location. The three main locations are highlighted in red.

Tables 1 and 2 provide comprehensive details regarding non-toric and toric IOL models used within our institution, excluding eight models with very low usage (1 or 2 lenses per year). They include specific data such as weight, packaging dimensions, manufacturing locations, shipping distances, estimated CO_2_ transport costs, and the number of IOLs implanted during 2023 at our institution. The information highlights significant variability in packaging weight (ranging from 37.5 to 142.3 g), packaging volume (from 135 to 917 cm³), and CO_2_ transport costs (ranging from 1.05 to 12.72 kg CO_2_ per 1,000 units). Manufacturing locations span the globe, as illustrated in the world map (Fig. 1).

**Table 1:** Specifications of Non-Toric Intraocular Lens Models Implanted in Our Institution in 2023.

	Manufacturer	IOL Model	Number of IOLs implanted	Box Weight (g)	Box Height (mm)	Box Width (mm)	Box Depth (mm)	Box Volume (cm³)	Manufacturing City	Manufacturing Country	Shipping Distance (km)	CO_2_ Transportation Cost per 1000 Units (kgCO_2_)
**One-piece**												
Alcon		Acrysof SN60AT	4	41.9	15.5	10.5	2.0	326	Cork	Ireland	943	1.06
		Acrysof SN60WF	68	–	–	–	–	-	-	-	-	-
		Acrysof IQ (**P,I**)	15	63.1	10.5	18.8	2.0	395	Fort Worth	USA	9572	7.08
		Clareon (**P,I**)	472	73.6	6.0	21.0	2.8	353	Cork	Ireland	943	1.85
B&L		Eye-cee One (**P,I**)	20	71.3	6.3	20.8	2.1	275	Aichi	Japan	20494	11.14
BVI		Carlevale FIL SSF*	58	38.5	10.8	8.5	3.2	294	Pontecorvo	Italy	4394	3.72
		Micropure	58	39.6	6.0	7.5	3.0	135	Liege	Belgium	363	1.08
Cristalens		Artis PL E (**P,I**)	3508	52.0	9.0	21.0	3.0	567	Lannion	France	846	1.55
Cutting Edge		Carlevale 2 (**P,I**)	15	95.2	8.5	19.0	5.0	808	Ahmedabad	India	11917	10.77
Hoya		XC1-SP (**P,I**)	1677	66.2	6.5	22.0	2.5	358	Lamphun	Thailand	15244	11.43
		XY1-SP (**P,I**)	1398	65.6	6.5	22.0	2.5	358	Lamphun	Thailand	15244	11.33
J & J		Tecnis (**P,I**)	888	83.3	23.0	10.5	2.5	604	Anasco	Puerto Rico	7120	5.89
Zeiss		Asphina 404 (**P**)*	5	–	–	–	–	-	-	-	-	-
		Asphina 509 (**P**)*	552	37.8	10.0	7.5	3.0	225	Guangzhou	China	17988	5.2
**Three-piece**												
Alcon		Acrysof MA50BM	16	43.8	15.5	10.5	2.0	326	Batam	Indonesia	15299	5.13
		Acrysof MA60AC	243	43.7	15.5	10.5	2.0	326	Batam	Indonesia	15299	5.12
		Acrysof MA60MA	44	44.0	15.5	10.5	2.0	326	Batam	Indonesia	15299	5.15
**Iris-claw**												
Cristalens		Artisan - Aphakia	105	39.3	12.0	9.3	2.2	246	Groningen	The Netherlands	1070	1.3
**Monofocals Plus**												
BVI		Isopure	623	40.8	6.0	7.5	3.0	135	Liege	Belgium	363	1.12
Hoya		Vivinex Impress (**P,I**)	236	66.0	6.5	22.0	2.5	358	Lamphun	Thailand	15244	11.4
J & J		Tecnis Eyhance (**P,I**)	2033	85.4	23.0	10.5	2.5	604	Anasco	Puerto Rico	7120	6.04
Rayner		EMV (**P,I**)*	106	142.3	9.3	19.4	3.5	631	Worthing	UK	445	2.92
**EDOF**												
Alcon		Vivity	65	44.2	15.5	10.5	2.0	326	Fort Worth	USA	9572	4.96
B & L		Luxsmart Yellow (**P,I**)	442	62.9	6.8	20.5	3.4	474	Montpellier	France	744	3.43
		Luxsmart Crystal (**P,I**)	11	–	–	–	–	–	–	-	-	-
J & J		Tecnis Symfony (**P,I**)	15	83.7	23.0	10.5	2.5	604	Anasco	Puerto Rico	7120	5.92
Medicontur		Elon (**P,I**)	5	–	–	–	–	–	–	-	-	-
**Multifocals**												
Alcon		Acrysof Panoptix	42	–	–	–	–	–	–	-	-	-
		Clareon Panoptix	28	42.2	15.5	10.5	2.0	326	Cork	Ireland	943	1.06
BVI		Finevision HP	3	–	–	–	–	–	–	-	-	-
		Finevision*	61	42.2	6.0	7.5	3.0	135	Liege	Belgium	363	1.15
Cristalens		Artis Symbiose Mid (**P,I**)	7	–	–	–	–	–	–	-	-	-
		Artis Symbiose Plus (**P,I**)	11	–	–	–	–	–	–	-	-	-
J & J		Tecnis Synergy (**P,I**)	6	–	–	–	–	–	–	-	-	-
Zeiss		AT Lisa Tri 839 (**P**)*	9	37.6	10.0	7.5	3.0	225	Perigny	France	1112	1.07

*B & L* Bausch & Lomb, *IOL* Intraocular lens, *I* with injector, *J & J* Johnson & Johnson, *P* Preloaded,* hydrophilic lens.

**Table 2:** Specifications of Toric Intraocular Lens Models Implanted in Our Institution in 2023.

Manufacturer	IOL Model	Number of IOLs implanted	Box Weight (g)	Box Height (mm)	Box Width (mm)	Box Depth (mm)	Box Volume (cm³)	Manufacturing City	Manufacturing Country	Shipping Distance (km)	CO_2_ Transportation Cost per 1000 Units (kgCO_2_)
**Monofocals**											
Alcon	Acrysof	81	44.9	15.5	10.5	2.0	326	Fort Worth	USA	9572	5.03
	Clareon	9	41.8	15.5	10.5	2.0	326	Cork	Ireland	943.0	1.05
	Clareon (**P,I**)	4	73.7	6.0	21.0	2.8	353	Cork	Ireland	943	1.86
B&L	Envista	25	87.5	13.0	7.7	3.8	380	Clearwater	USA	8126	6.23
	Envista (**P,I**)	71	133.8	21.0	7.4	5.9	917	Rochester NY	USA	6504	12.72
BVI	Ankoris*	21	41.3	6.0	7.5	3.0	135	Liege	Belgium	363	1.13
	Podeye	10	–	–	–	–	–	–	-	-	-
Cristalens	Artis (**P,I**)	218	52.3	9.0	21.0	3.0	567	Lannion	France	846	1.56
Hoya	Vivinex (**P,I**)	78	65.4	6.5	22.0	2.5	358	Lamphun	Thailand	15244	11.29
J&J	Tecnis II	14	90.8	16.3	11.1	1.9	344	Anasco	Puerto Rico	7120	6.42
Zeiss	AT Torbi 719 (**P**)*	56	37.5	10.0	7.5	3.0	225	Berlin	Germany	1763	1.82
**Monofocals Plus**											
J&J	Eyhance (**P,I**)	349	83.7	23.0	10.5	2.5	604	Anasco	Puerto Rico	7120.0	5.9
Rayner	EMV (**P,I**)*	4	142.0	9.3	19.4	3.5	631	Worthing	UK	445	2.91
**EDOF**											
Alcon	Vivity	29	–	–	–	–	–	–	-	-	-
B&L	Luxsmart (**P,I**)	24	62.8	6.8	20.5	3.4	474	Montpellier	France	744	3.43
**Multifocals**											
Alcon	Acrysof Panoptix	5	–	–	–	–	–	–	-	-	-
	Clareon Panoptix	8	42.3	15.5	10.5	2.0	326	Cork	Ireland	943	1.07
BVI	Finevision*	4	–	–	–	–	–	–	-	-	-
Cristalens	Artis symbiose Plus (**P,I**)	8	–	–	–	–	–	–	-	-	-
Zeiss	AT Lisa Tri 949 (**P**)*	12	–	–	–	–	–	–	-	-	-

*B&L* Bausch & Lomb, *IOL* Intraocular lens, *I* with injector, *J&J* Johnson & Johnson, *P* Preloaded, * hydrophilic lens.

**Fig. 2:**
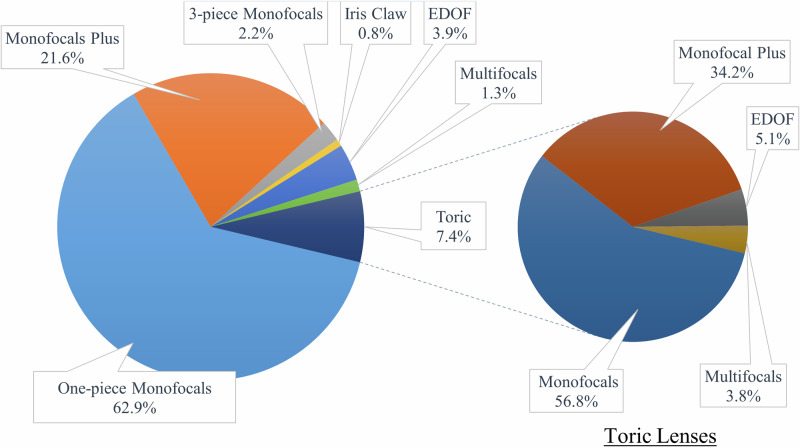
On the left, a pie chart depicting the distribution of the 13,894 intraocular lenses implanted at our institution in 2023. On the right, a nested pie chart illustrates a subanalysis focusing specifically on toric lenses.

In 2023, a total of 13,894 IOLs were implanted at our institution by a team of 112 ophthalmic surgeons, including 69 anterior segment specialists, 34 retina specialists and 9 paediatric specialists. The predominant IOL type was the standard monofocal, with 9,150 lenses (65.9%) implanted, including 305 three-piece monofocal IOLs (2.2%). Additionally, 2,998 monofocal “plus” lenses (21.6%) were implanted. A smaller number of toric lenses totalled 1,033 implants (7.4%). Furthermore, 538 non-toric EDOF lenses (3.9%) and 175 non-toric multifocal lenses (1.3%) were also implanted (Fig. 2).

**Fig. 3:**
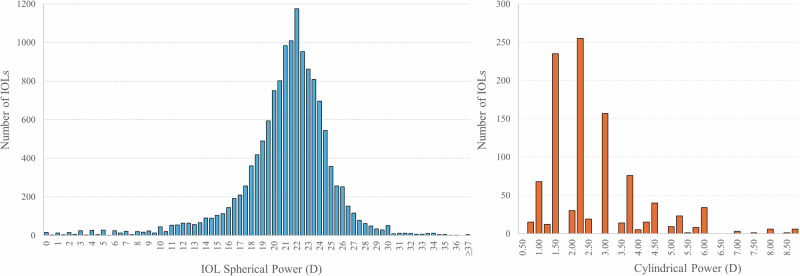
The bar chart on the left illustrates the distribution of spherical lens powers across all intraocular lenses (13,894 lenses). On the right, the bar chart depicts the distribution of cylindrical powers specifically for toric lenses (1033 lenses).

Figure 3 illustrates the distribution of spherical power among all implanted lenses and cylindrical power among toric lenses. The spherical power distribution forms a bell-shaped leptokurtic curve, with the 22.0D lens being the most common, accounting for 8% of the implants. Within the range of ± 2.0 dioptres around 22.0D, 58% of the implants are distributed.

The most prevalent cylindrical power among toric lenses is 2.25D, constituting 25% of all toric lenses implanted. Together, the cylindrical powers of 1.5D, 2.25D, and 3.0D account for 63% of all toric lenses implanted.

IOL models can be delivered in three packaging configurations: preloaded into an injector, preloaded without an injector (requiring a separate single-use injector), or not preloaded (requiring a reusable sterilisable injector). In our study, 84% of IOL models were preloaded into an injector, 5% were preloaded without an injector, and 11% were not preloaded. The weights of the packaging for each category were 81.2 ± 26.7 g, 37.6 ± 0.1 g, and 47.6 ± 15.3 g, respectively, with corresponding volumes of 514 ± 164 cm³, 225 ± 0.0 cm³, and 278 ± 83.4 cm³.

Nearly all IOLs utilised at our institution were hydrophobic, with hydrophilic lenses comprising only 6%. Additionally, our findings highlighted variations in surgeon preferences regarding UV yellow filters for Hoya monofocal lenses. These lenses are available with or without the filter stored together in the pharmacy inventory, offering surgeons flexibility in their choice. Approximately 45% of the implanted Hoya monofocal lenses included the yellow filter, indicating a lack of consensus among surgeons on this matter.

## Discussion

This study investigated the ecological impact associated with transporting IOLs from manufacturing sites to our institution, revealing significant variability in CO2 equivalent emissions among different IOL models. The observed variability, ranging from 12.72 kg CO2 for 1000 units to 1.05 kg CO2, indicates a 12-fold difference and underscores the environmental implications related to packaging size and shipping distances. These findings underscore the importance of optimising packaging strategies to reduce the ecological footprint of IOL distribution.

The transport footprint of IOLs is influenced by the global dispersion of manufacturing sites (see Fig. 1) and inefficient packaging strategies. Shipping distances vary widely depending on each institution’s location, posing challenges for a comprehensive global evaluation of this factor. Larger international companies could potentially reduce transport emissions by promoting local markets and aligning production with distribution locations. However, many manufacturers segregate manufacturing sites based on IOL models rather than proximity to markets, citing technical considerations.

Moreover, a notable trend among Western manufacturers is to relocate production to lower-cost regions, as observed in certain studied IOL models. This policy inadvertently increases CO_2_ emissions related to transport. Therefore, surgeons should consider that choosing local brands may not necessarily reduce ecological footprints.

The significant variability in packaging weight and volumes highlights that some manufacturers employ inefficient packaging practices, resulting in increased waste of raw materials and environmental costs during IOL transport. The largest IOL packaging volume (917 cm³) is approximately seven times larger than the smallest packaging (135 cm³).

The practice of oversized packaging primarily arises from marketing strategies aimed at enhancing perceived value rather than practical necessity. Manufacturers should prioritise minimising packaging size rather than artificially inflating it. Furthermore, reductions in package volume can be achieved by eliminating unnecessary items such as large paper Instructions for Use (IFU) brochures and promoting lighter, less voluminous electronic alternatives [4–6]. Surgeons should consider the environmental impact of packaging when selecting implants, favouring those with eco-friendly packaging.

Another factor contributing to increased packaging size and weight is the preloading of IOLs into single-use injectors. Models that include injectors within the packaging have an average volume nearly double that of non-preloaded models (514 cm³ versus 278 cm³).

While preloaded IOLs in injectors streamline surgeries and reduce intraoperative complications associated with IOL loading, they simultaneously increase packaging dimensions and especially the packaging weight of hydrophilic lenses, as the entire injector needs to be in water. This technology appears to be highly appreciated by surgeons, as evidenced by 89% of the implanted IOLs in our study being preloaded. However, it appears that this technology is not environmentally friendly, generating significant plastic waste. A greener alternative approach, already offered by some manufacturers though not currently available at our institution, would be to use preloaded IOLs in cartridges with reusable injectors. This approach would promote sustainability by reducing raw material waste and transport costs associated with preloading technology.

As ophthalmologists, we must remain aware of environmental concerns, as the healthcare sector accounts for nearly 10% of global greenhouse gas emissions [3], making its ecological footprint impossible to overlook. One particularly critical area requiring attention is the operating theatre, which generates up to 30% of a hospital’s total waste [7].

Cataract surgery is the most frequently performed procedure in ophthalmology [8], with approximately 20 million surgeries conducted worldwide each year [9, 10]. Its environmental impact is substantial, with each procedure producing an estimated 180 kg of CO₂—nearly half of which is attributed to the procurement of medical equipment and pharmaceuticals [11]. Additionally, while IOL packaging may appear negligible, it contributes approximately 7.4% of the total waste generated by cataract surgeries [12].

The impact of oversized packaging extends beyond environmental concerns, affecting the storage efficiency of operating room pharmacies, which are typically confined spaces. Efficient inventory management could profoundly influence surgical practices by ensuring a supply of premium lenses, such as toric, EDOF, and multifocal IOLs, readily available for surgeons without requiring special orders. This increased availability could boost the utilisation of premium lenses, which currently account for only 12.6% of implanted IOLs at our tertiary care centre. Surgeons would have the flexibility to select a premium lens at the last minute prior to surgery.

Promoting premium lenses is crucial, especially considering our findings, which indicate that surgeons predominantly use standard monofocal lenses (approximately 70%). This reflects a conservative approach and a reluctance to adopt premium lenses. In many instances, this reluctance is justified by logical and clinical considerations, as premium lenses can introduce optical aberrations or diffraction into the eye, potentially reducing visual quality or causing photic phenomena. These concerns are particularly problematic in cases of compromised ocular health, such as glaucoma or macular diseases, which are frequently encountered in specialised centres like ours. However, in some cases, surgeons opt for monofocal lenses simply because they are easier to manage.

Interestingly, we observed that monofocal “plus” lenses, which are consistently stocked and available in all powers as monofocal options at our institution, were implanted in more than 20% of cases. This trend largely arises from their popularity as monofocal lenses that provide high optical quality and slightly enhanced depth-of-focus, are compatible with other concomitant ocular diseases, are readily available for surgeons, and do not incur additional costs for patients.

The low utilisation rate of toric lenses (7.4%) is particularly perplexing given that corneal astigmatism greater than 1.5D affects 16–26% [13–15] of the population. This underuse cannot be attributed solely to contraindications such as pseudoexfoliation, zonulopathy, or small pupil size [16], nor to alternative astigmatism treatment methods like manual or femtosecond laser incisions [17], which are infrequently employed in our hospital. This low utilisation rate of toric lenses represents a missed opportunity to optimise visual outcomes.

This observation aligns with a retrospective study of 282,811 cataract cases conducted from 2014 to 2015 using data from the EUREQUO database, which reported inadequate management of astigmatism during cataract surgery, with approximately 30% of patients experiencing residual astigmatism exceeding 1 dioptre post-surgery [18]. Market share data further indicate that toric IOLs are implanted in only 10–15% of cases in most developed countries [19], highlighting that toric IOLs have not yet become the standard-of-care for astigmatic patients despite their proven benefits.

Toric lenses impose an additional workload and time on surgeons during surgery, requiring precise placement in the eye after identifying the correct positioning using either manual or automatic methods. Another frequently cited reason for the non-use of toric lenses is patient reluctance due to the additional cost involved, as toric lenses are considered premium options. However, patients should be informed that opting for monofocal lenses may result in increased post-surgery expenses due to the potential necessity for multifocal toric glasses, which are more expensive compared to multifocal glasses without astigmatism correction [20, 21].

As previously mentioned, toric lenses, as well as all premium lenses, also impose an additional preoperative workload, requiring special orders and ensuring the lens for the patient is available. Optimising the management of IOL inventory could facilitate the stocking of toric lenses for regular availability alongside monofocal lenses. Our data show that most implanted lenses fall within a range of ± 2D of 22.0D (58%). Additionally, only three different cylinder powers of toric lenses are needed to cover 63% of astigmatic patients, demonstrating that efficient management of IOL inventory would allow for the availability of premium lenses for the majority of patients.

Improved IOL stock management can also contribute to reducing CO₂ emissions associated with transport. We recommend that manufacturers minimise excessive packaging by adopting smaller, more efficient designs. Compact packaging would allow for increased storage capacity, potentially facilitating the routine availability of toric and premium IOLs alongside monofocal lenses. This change could mitigate the high CO₂ emissions currently generated by special-order shipments from distribution centres. Additionally, we advocate for the implementation of sustainable transport methods, such as utilising electric vans instead of motorcycle couriers for special deliveries.

From a consumer perspective, ophthalmologists should be mindful of the CO₂ cost of transport. Surgeons are encouraged to enquire about the manufacturing locations of IOLs and to prioritise products manufactured closer to their practice sites. Supporting local production can help mitigate the environmental impact associated with the relocation of manufacturing facilities.

A feasible, albeit challenging, strategy to further reduce transport-related CO₂ emissions is to streamline the variety of IOLs used. By standardising a select range of lenses and maintaining larger inventories of these standardised products, surgical centres could minimise the need for special orders of premium IOLs, thereby further decreasing the carbon footprint associated with transport. Our study has several limitations. We assumed that packaging measurements and manufacturing sites remained consistent between 2023 and May 2024. Additionally, we did not account for multiple manufacturing sites, intermediate storage locations, or inefficient transport, all of which substantially impact transport-related emissions. As outlined in the Methods section, our estimation represents the minimum possible CO₂ emissions. Moreover, the shipping distances were calculated for Paris, France, where our hospital is located, and these distances vary significantly in different locations around the world. Furthermore, our pharmacy stock software did not identify the rare cases where multiple IOLs were used for the same patient or research purposes.

In conclusion, variability in CO2 emissions from IOL transport among different models suggests that some manufacturers could adopt greener practices by reducing packaging size, refraining from relocating manufacturing sites, and promoting local production. Improving IOL stock efficiency through smaller packaging could also enhance the availability and utilisation of premium lenses, which are currently underutilised.

## Summary

### What was known before

- The healthcare industry is responsible for approximately 10% of overall greenhouse gas (GHG) emissions in the United States. Transportation of medical supplies is one of the elements contributing to GHG emissions and needs to be addressed.
- The intraocular lens (IOL) market is a global industry with products sourced worldwide. It is highly competitive, and manufacturers often use large packaging as a marketing strategy to influence surgeons’ choices, who may be hesitant to adopt new IOLs.

### What this study adds

- The carbon footprint of IOL transportation varies significantly between models. Optimising packaging and using reusable injectors could reduce this footprint and stock volume, enhancing the availability of premium implants without the need for special orders. This could potentially increase the use of premium lenses, particularly toric lenses, which should be the gold standard for patients with astigmatism.
- Monofocal lenses remain the gold standard, accounting for about 70% of surgeons’ choices, while monofocal “plus” lenses have a surprisingly high popularity at 20%. Premium lenses, including toric (7.4%), extended depth-of-focus (EDOF) (3.9%), and multifocal lenses (1.3%), are used less frequently than anticipated.

## Data Availability

The data supporting the findings of this study are not publicly available due to confidentiality restrictions. However, they can be made available upon reasonable request by contacting the corresponding author.
